# Multiple-scale spatial analysis of paediatric, pedestrian road traffic injuries in a major city in North-Eastern Iran 2015–2019

**DOI:** 10.1186/s12889-020-08911-2

**Published:** 2020-05-19

**Authors:** Hamidreza Shabanikiya, Soheil Hashtarkhani, Robert Bergquist, Nasser Bagheri, Reza VafaeiNejad, Malihe Amiri-Gholanlou, Toktam Akbari, Behzad Kiani

**Affiliations:** 1 Social Determinants of Health Research Centre, Mashhad University of Medical Sciences, Mashhad, Iran; 2 Department of Medical Informatics, Faculty of Medicine, Mashhad University of Medical Sciences, Mashhad, Iran; 3 Ingerod, Brastad, Sweden (formerly with the UNICEF/UNDP/World Bank/WHO Special Programme for Research and Training in Tropical Diseases, World Health Organization), Geneva, Switzerland; 4 Visualization and Decision Analytics (VIDEA) lab, Centre for Mental Health Research, Research School of Population Health, College of Health and Medicine, The Australian National University, Canberra, Australia; 5 Center for Accident and Emergency Medicine Management, Mashhad University of Medical Sciences, Mashhad, Iran; 6 Student Research Committee, School of Health, Mashhad University of Medical Sciences, Mashhad, Iran

**Keywords:** Spatial analysis, Geographical information system, Paediatric, Pedestrian accident, Road traffic injuries, Iran, Cluster analysis

## Abstract

**Background:**

Paediatric, pedestrian road traffic injuries (PPRTIs) constitute a major cause of premature death in Iran. Identification of high-risk areas would be the primary step in designing policy intervention for PPRTI reduction because environmental factors play a significant role in these events. The present study aims to determine high-risk areas for PPRTIs at three different geographical scales, including the grid network, the urban neighbourhood and the street levels in Mashhad, Iran during the period 2015–2019.

**Methods:**

This cross-sectional retrospective study was based on all pedestrian accidents with motor vehicles involving children (less than 18 years of age) between March 2015 and March 2019 in the city of Mashhad, which is the second-most populous city in Iran. The Anselin Local Moran’s *I* statistic and Getis-Ord Gi* were performed to measure spatial autocorrelation and hotspots of PPRTIs at the geographical grid network and neighbourhood level. Furthermore, a spatial buffer analysis was used to classify the streets according to their PPRTI rate.

**Results:**

A total of 7390 PPRTIs (2364 females and 4974 males) were noted during the study period. The children’s mean age was 9.7 ± 5.1 years. Out of the total PPRTIs, 43% occurred on or at the sides of the streets, 25 of which labelled high-risk streets. A high-high cluster of PPRTI was discovered in the eastern part of the city, while there was a low-low such cluster in the West. Additionally, in the western part of the city, older children were more likely to become injured, while in the north-eastern and south-eastern parts, younger children were more often the victims.

**Conclusions:**

Spatial analysis of PPRTIs in an urban area was carried out at three different geographical scales: the grid network, the neighbourhood and the street level. The resulting documentation contributes reliable support for the implementation and prioritization of preventive strategies, such as improvement of the high-risk streets and neighbourhoods of the city that should lead to decreasing numbers of PPRTIs.

## Background

Up to 22% of all road fatalities across the world involve pedestrians [1]. Children are the most vulnerable group among pedestrians due to their less developed cognitive and physical status [2]. Various factors play a role in accidents implicating paediatric pedestrians, which can be divided into the two general categories with regard to the cause: personal and environmental factors [3–5], where the latter is the most important. This is also a reflection of a report by the *World Health Organization* (WHO) on *Road Traffic Injuries* (RTIs) that emphasises that the main risk factors for pedestrian accidents are due to the immediate environment [5]. These factors include traffic volume, population density, urban design, socioeconomic status and, importantly, the existence or non-existence of safe passageways [5–7]. Thus, it seems that the primary steps in designing any intervention to reduce the number of accidents would be the identification of high-risk locations where the environmental factors can be characterized and documented in a way that leads to the implementation of preventive strategies.

In Iran, the number of road traffic deaths fell from 32 per 100,000 in 2015 to 20 per 100,000 in 2018. However, the impact of road traffic casualties is still very high, with nearly 17,000 people killed, and more than 351,000 injured as a result of traffic accidents every year [8]. Indeed, RTI is the fourth cause of death in all age groups and is the second cause of premature death in Iran [9], resulting in 1367 *Disability-Adjusted Life Years* (DALYs) lost per 100,000 in 2017 [10]. Compared to other provinces of the country, Khorasan-Razavi ranks second in terms of road traffic accidents, and 76% of all traffic casualties occurred in the provincial capital, the city of Mashhad [11, 12]. With about a million people in the suburbs and a total population of 3,372,660, Mashhad has the largest suburban population in Iran [13], which translates into a large population of poor children [13]. Moreover, being a child, or a member of a low-income family, are two known risk factors for pedestrian accidents [14–16]. *Paediatric, Pedestrian Road Traffic Injuries* (PPRTIs) is, therefore, an important and crucial public health issue for this city, whose reduction would require effective interventions. According to the WHO guideline for pedestrian safety, the first step with respect to prioritizing interventions and preparing an action plan relates to assessing the pedestrian safety situation. Examining the places where pedestrian accidents occur is one of the items that needs to be considered [1]. A safety situational analysis of the key areas of Mashhad’s PPRTIs is critical with regard to developing an action plan for this city. The results should identify high-risk locations for these kinds of accidents and reveal attributes and characteristics of such places, an activity that can be generalized for the benefit of highly populated cities in general.

So far, only a few studies on the subject of PPRTIs have been carried out. For example, a case-control study by Pernica et al. [17] conducted to investigate the relationship between general population, family and travel factors involving PPRTIs in Lima, Peru. The findings of this study indicated that factors, such as parental education level, duration of outside play, supervision during outside play (or the lack thereof) and the number of the streets needed to be crossed when walking to school are associated with PPRTIs. In another study, Tetali et al. [18] did a cross-sectional survey on 5789 students aged 11–14 years in Hyderabad, India to identify and estimate the prevalence of variables related to road injuries taking place on route to school. Their results showed that children who cycled to school were more likely to suffer injuries compared to children who walked; however, the children who walked to school were more likely to be injured compared to those who went to school by school bus. On the other hand, these two studies did not examine where the injuries took place, thus leaving definition of a high-risk places of PPRTIs to future research.

Most researchers have used *Geographical Information Systems* (GIS) to identify PPRTI high-risk places. GIS enables researchers to perform spatial analyses of accident data [19]. Such analyses seek to explain environmental exposure and geographical factors based on the spatial pattern of human behaviour, [20, 21]. By combining spatial and non-spatial data [22], GIS contribute to enabling researchers to analyse patterns in more detail, e.g., focusing on PPRTIs. In this vain, Curtis et al. [23] used a combination of different geospatial analytical methods, including proximity analysis, kernel density estimation and local Moran’s *I* to examine the geographical distribution of pedestrian injuries in a mid-size city in the United States, while Lightstone et al. [24] conducted a study in Long Beach, California to locate high-risk areas for children being hit by motor vehicles. The study showed that 65.6% of such accidents occurred on local and so-called collector streets, which are less than 35 ft (10.7 m) wide. Despite several similar studies around the world no such study, to the best of our knowledge, has been undertaken in Iran. Furthermore, previous studies mostly conducted in mid-size cities of industrialized countries. The analyses of such studies dealt with one geographical scale only. These cities are different than large cities in developing countries. There is limited knowledge of high-risk locations for PPRTIs in a largely populated city in the context of a developing country such as Iran. The present study aimed to determine high-risk areas for PPRTIs at three different geographical scales including, the grid network, the urban neighbourhood and the street levels in Mashhad, Iran during the period 2015–2019.

## Methods

### Study area

This study was conducted in the city of Mashhad, the capital of Khorasan-Razavi Province and the second-most populous city in Iran. It is located in north-eastern Iran and has a population of 3,372,660 according to the 2016 national census. The average annual income of urban households of Khorasan-Razavi Province in 2018 was $7523 [25] and Gini Coefficient for urban areas of the province in 2018 was 0.3802 [26].

### Data source and geocoding

We used the *Emergency Medical Services* (EMS) database of the city of Mashhad. Data related to emergency care calls in Mashhad between March 2015 and March 2019 were extracted and processed further to obtain the PPRTIs. An emergency mission is an ambulance service to the scene of a child’s accident with a vehicle, and if necessary, the child is transported to the hospital. The records of the emergency database had a textual data field explaining the emergency mission. One of the authors read the content of that field for every record and marked the records related to PPRTIs.

Due to the lack of standard tools for geocoding Persian addresses, we geocoded all addresses manually by searching each address on Google Maps. Prior studies have shown that the quality of Google’s data is higher than some other geocoding tools such as R packages and GIS [27, 28]. Google Maps are defined and owned by Google. But, Google’s MyMaps (http://www.google.com/mymaps) is a free tool developed by Google for users to create their own maps. This latter application was used for geocoding the addresses, which were then exported to a *Keyhole Markup Language* (KML) file and imported into ArcGIS 10.6 software [29] for further spatial analysis.

### Ethical approval

The study was approved by the ethical committee of Mashhad University of Medical Sciences with a reference number of 970,733.

### Spatial analysis

Hotspots and outliers play an essential role in visualizing and quantifying geographic variation patterns. Hotspot areas have statistically significant more PPRTIs in comparison to the neighbouring regions and the whole study area. There are two important types of spatial statistics to identify geographical variations in the PPRTI rate; global cluster statistics and local spatial statistics. The global methods (e.g., K-function, Cuzick Edwards, Kernel density estimation, and Global Moran’s *I*) are more sensitive to departures from the null hypothesis, which assumes that PPRTIs are randomly distributed in the area under study. Although they can identify spatial structures, they do not determine where the clusters are [30]. Local cluster statistics (e.g., Anselin’s Local Moran’s *I* and Getis-Ord Gi*) quantify spatial autocorrelation and clustering at the small area level [31]. As this study aimed to detect both spatial variation and spatial clusters, Anselin’s local Moran’s *I* and Getis-Ord Gi* were used.

Local Moran’s *I* statistic calculates *Z*-score and *p*-value to indicate whether the apparent similarity, i.e. spatial clustering, expressed as either high-high (HH) or low-low (LL) or dissimilarity (expressed as either high-low (HL) or low-high (LH) values), is more pronounced than one would expect for a random distribution. The null hypothesis states that PPRTIs are randomly distributed across the study area [32]. Thus, HH and LL regions are target areas surrounded by areas with similar PPRTI rates, while for HL and LH regions, the target areas are surrounded by areas with dissimilar PPRTI rates [30]. In other words, the HH and LL indicate clusters, while the HL and LH indicate outliers.

The Getis-Ord Gi* statistc shows where features with either high or low values cluster spatially and identifies each feature within the context of neighbouring features [33]. A feature is a geographical unit, for example, a neighbourhood. A feature with a high value is interesting, but may not be a statistically significant hotspot. To be statistically significant, such a feature would have to show a high value surrounded by other features with high values. This statistic is given as:

1 $$ {G}_i^{\ast }=\frac{\sum_{j=1}^n{w}_{i,j}{x}_j-\overline{\mathrm{X}}{\sum}_{j=1}^n{w}_{i,j}}{\sqrt[s]{\frac{\left[n{\sum}_{j=1}^n{w}_{i,j}^2-{\left({\sum}_{j=1}^n{w}_{i,j}\right)}^2\right]}{n-1}}} $$

where the ${G}_i^{\ast }$ statistic is the *Z*-score; x_j_ the number of PPRTIs for the feature *j*; *w*_*i,j*_ the spatial weight between feature *i* and *j*; while *n* is equal to the total number of features and

2 $$ \overline{X}=\frac{\sum_{j=1}^n{x}_j}{n} $$

3 $$ S=\sqrt{\frac{\sum_{j=1}^n{x}_j^2}{n}-{\overline{X}}^2} $$

### Statistical approach

#### The geographical grid network level

A geographical grid network is a system of imaginary arcs that divide Earth’s surface into latitude and longitude. Anselin Local Moran’s *I* was used to identify clusters and outliers of PPRTIs at this level, i.e. covering to the whole city. Furthermore, we used Getis-Ord Gi* to perform the hotspot analysis by considering the children’s ages in the analysis field. Analysing point accidents with an analysis field allows researchers to answer questions such as where do high and low values cluster, which means that we could find all the clusters with high-age PPRTIs and low-age PPRTIs across the study area. High-age areas have a significantly higher mean age of children’s accidents, but low-age areas have a significantly lower mean age of children’s accidents.

#### The neighbourhood level

In addition to cluster analysis at the geographical grid network level, we also defined the urban neighbourhood as a geographical boundary, including all 73 neighbourhoods in the study area. The Anselin - Local Moran’s *I* statistic was used again to determine the clusters and outliers of PPRTIs at this level in the city of Mashhad.

#### The street level

After obtaining the street vector layer of the city from Mashhad Municipality, a 10-m buffer for every street was created and the spatial join tool used to determine the number of PPRTIs for each street. We were then able to calculate the PPRTI rate per 100,000 people accounted along the length of each street. This rate (number of PPRTIs to street length) was used for street classification. The natural break method with four classes was used to classify this index [34]. The streets of the first, second, third and fourth grade were identified as low-risk, low-intermediate risk, intermediate-high risk and high-risk streets, respectively.

The *World Geodetic System* (WGS) is a geographic coordinate system based on a spheroid and utilizes angular units (degrees). The latest revision is WGS 84 (also known as WGS 1984). *Universal Transverse Mercator* (UTM) System is a projected coordinate system based on a plane (the spheroid projected onto a 2D surface) and utilizes linear units (feet, meters, etc.). In this study, all layers were projected to WGS_1984_UTM_Zone_40N. Projection is a mathematical transformation that transforms spherical coordinates (latitude and longitude) into an XY (planar) coordinate system. This enables researchers to create a map that accurately shows distances, areas, or directions. WGS_1984_UTM_Zone_40N is suitable for use between 54°E and 60°E, and in the northern hemisphere between the equator and 84°N, onshore and offshore. We used this projection system because the study area is located around 59°E and 36°N.

The spatial analyses were performed in ArcGIS 10.6 [29], and the descriptive analysis was conducted by Microsoft Excel 2016.

### Statistical significance

The Gi* statistic and Anselin local Moran’s *I* calculate a z-score and *p*-value for each feature in the dataset. *P*-value and z-score are associated with each other. Here p-value means the probability that the identified spatial pattern of a feature is due to chance. Using the Gi* statistic, high positive z-scores show the clustering of high values (hotspot), and low negative z-scores show the clustering of low values (coldspot). For Anselin Local Moran’s *I*, A high positive z-score for a feature indicates that the surrounding features have similar values (either high values or low values). However, a low negative z-score for a feature indicates a statistically significant spatial data outlier [35]. We used a 95% *Confidence Level* (CL), and all clusters and outliers found in this study were significant at this CL.

## Results

**Fig. 1:**
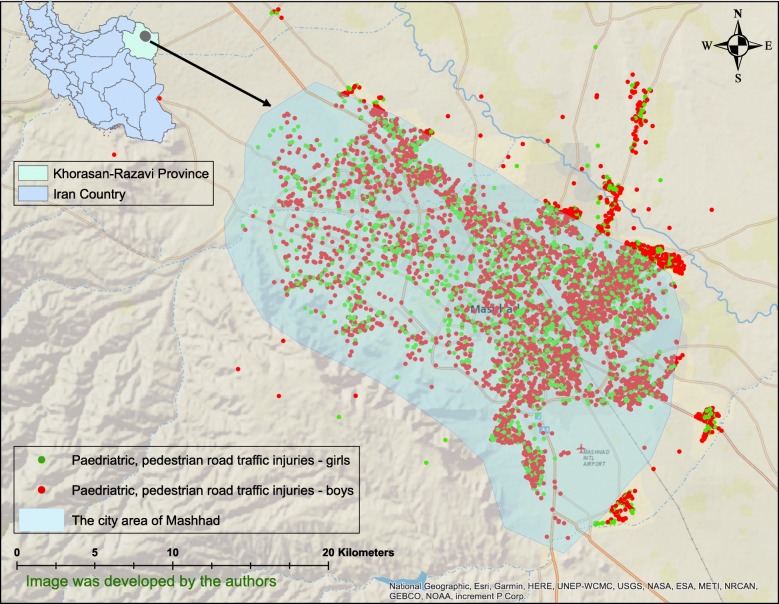
Geographical distribution of paediatric, pedestrian road traffic injuries in the city of Mashhad, Iran in the period 2015–2019

**Fig. 2:**
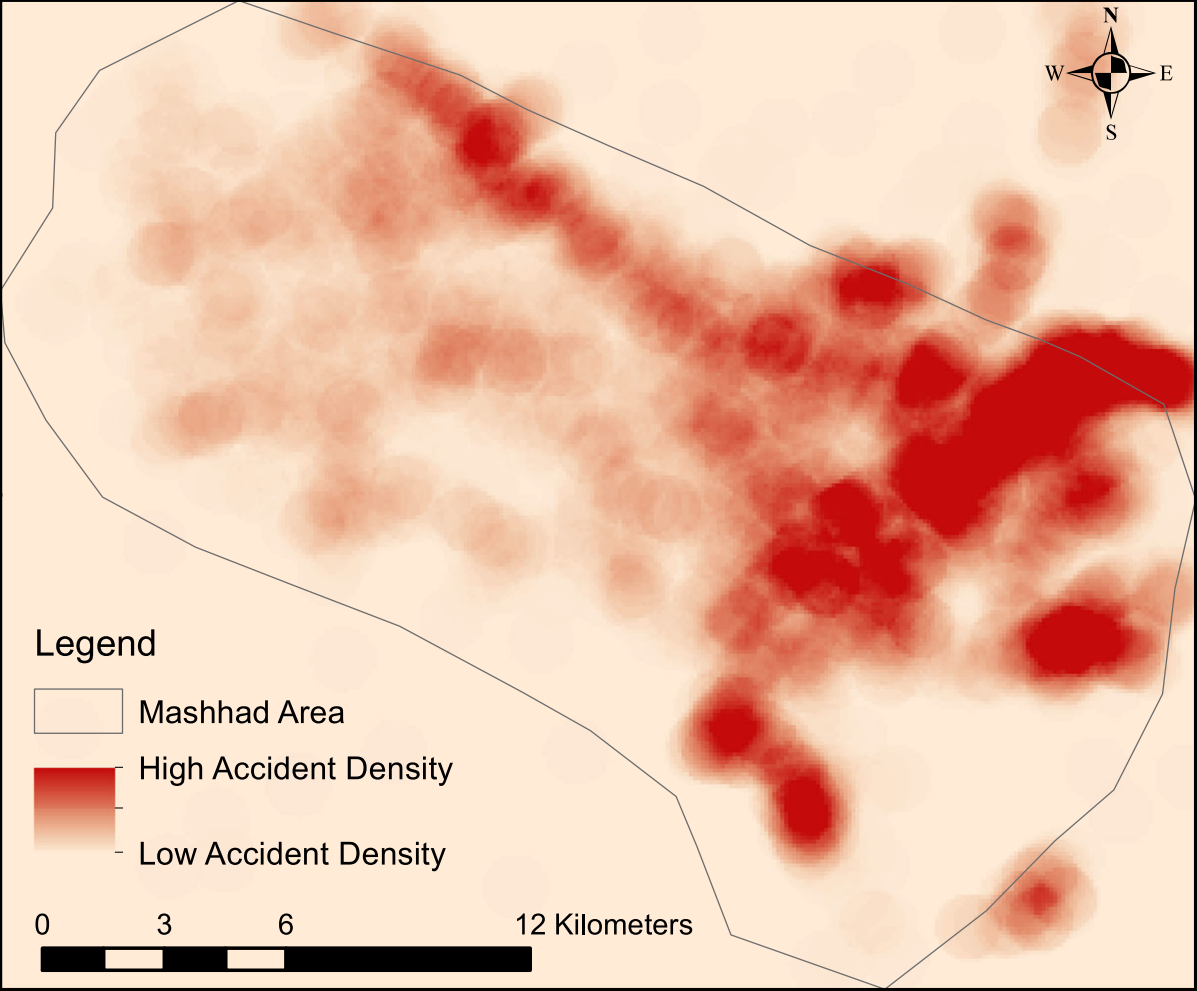
Point density map of paediatric, pedestrian road traffic injuries in the city of Mashhad, Iran in the period 2015–2019

A total of 7390 PPRTIs consisting of 2364 girls and 4974 boys (however, the data did not allow the gender to be recorded for 52 cases) were found in the city of Mashhad in the period covering March 2015 to March 2019 (Fig. 1). The children’s mean age was 9.7 ± 5.1 years. Figure 1 shows the location of the study area and the geographical distribution of PPRTIs, while Fig. 2 shows the density of the accidents in the study area.

**Fig. 3:**
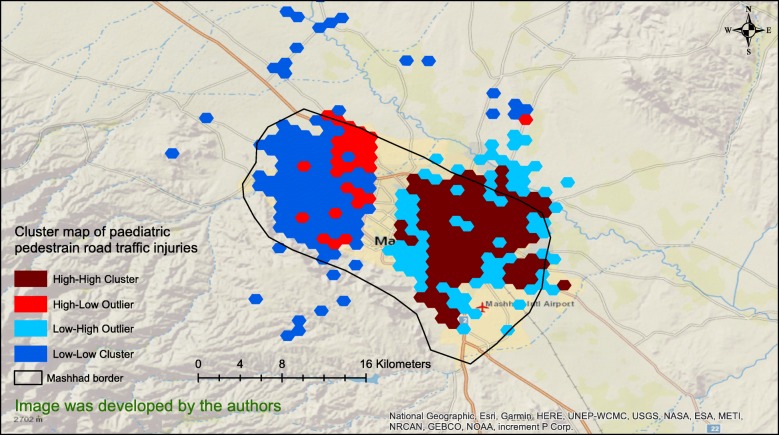
Clustered paediatric, pedestrian road traffic injuries in the city of Mashhad, Iran in the period 2015–2019

Figure 3 reveals the results of cluster analysis of the PPRTIs at the geographical grid network level. It clearly shows a HH cluster in the eastern part of the city separated from a LL one in the western part by a band through the middle of the city. In addition, there was a HL outlier in the north-western part of the study area (Fig. 3). All clusters and outliers were statistically significant (*P* < .05).

**Fig. 4:**
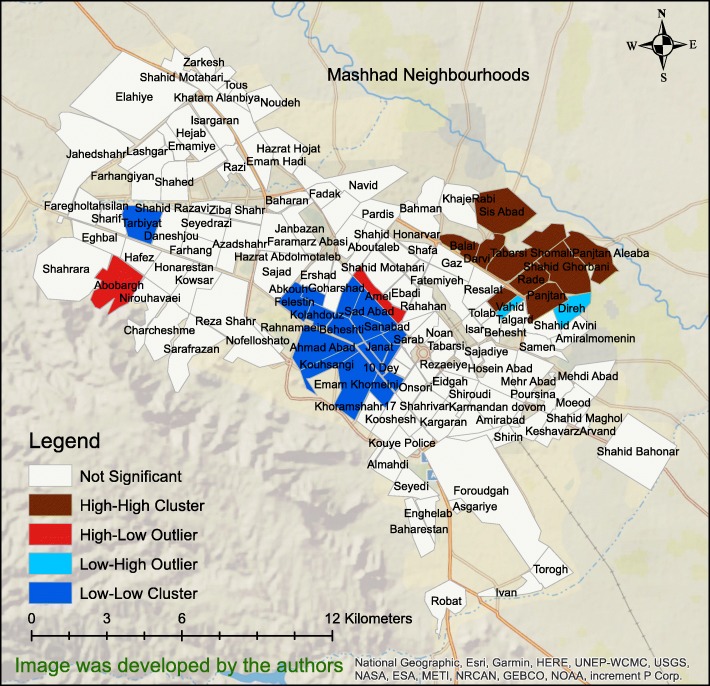
The results of Local Moran’s I statistic of the paediatric, pedestrian road traffic injuries at Mashhad neighbourhood level

Figure 4 shows the result of Anselin - Local Moran’s *I* statistic at neighbourhood level of the city of Mashhad. As the figure shows, there were HH clusters in the north-eastern part of the city, some LL clusters in the middle of the city and two HL outliers in the Abobargh and Ameli neighbourhoods. All clusters and outliers were statistically significant (*P* < .05).

Getis-Ord Gi* has an option for analysing the PPRTIs. Researchers can set the option with one of the data fields as ‘Analysis Field’ or leave it. If left blank, it would allow researchers to identify where point clustering is unusually intense or sparse, i.e. statistically significant. However, analysing PPRTI points with an ‘Analysis Field’ allows researchers to answer the question ‘Where do high and low values cluster?’

**Fig. 5:**
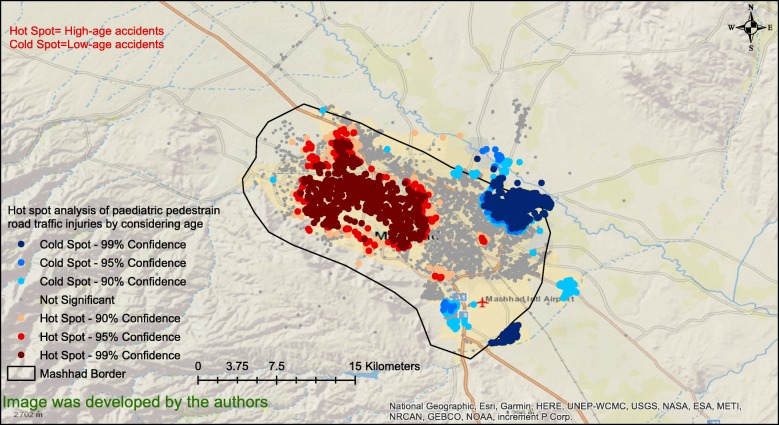
Hotspot analysis of paediatric, pedestrian road traffic injuries by considering age in the city of Mashhad Iran, in the period 2015–2019

To determine if there was a spatial pattern in the age of the children who experienced accidents, the hotspot analysis of PPRTIs was conducted considering the child age for the analysis. Figure 5 clearly shows that in the western part of the city, older children are more likely to be involved in accidents. However, in the north-eastern and south-eastern parts of the city, younger children are the more common victims. In this figure, coldspots show the regions where the average age of children’s accidents was significantly low, and also surrounded by low-age areas. However, hotspots show the regions where the average age of children’s accidents was significantly high, and also surrounded by high-age areas.

**Fig. 6:**
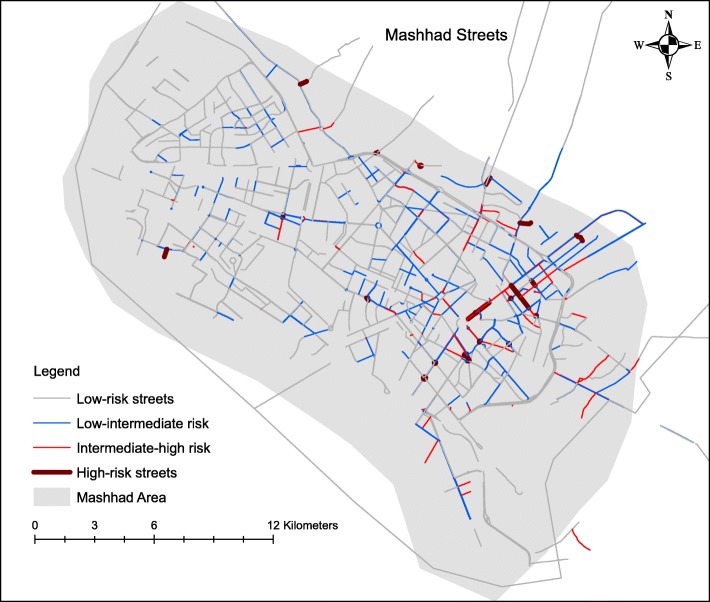
Street risk classification based on paediatric, pedestrian road traffic injuries in the city of Mashhad Iran, in the period 2015–2019

Figure 6 shows the street risk classification based on PPRTIs. There are 2227 streets in the city of Mashhad and 43% of the PPRTIs occurred on the street or along their sides. We identified 643 streets as low-risk ones, 307 as low- intermediate risk ones, 105 as intermediate-high risk ones and 25 as high-risk ones, while 1139 streets were completely free from accidents.

## Discussion

Our results show that the majority of injured paediatric pedestrians were boys, which is in accordance with the results of a similar study of children brought to a tertiary paediatric referral trauma department in Cape Town, South Africa between 2004 and 2013 [36]. Additionally, some other studies have found that the majority of injured paediatric pedestrians were boys [37, 38]. It seems that girls generally behave more safely than boys. O’Neal et al. [4] assessed the behaviour of crossing the road by designing a road-crossing act in a virtual environment. The results of their study revealed that girls were more cautious than boys when crossing the virtual roadway.

Interestingly, the HH cluster in the eastern and the LL cluster in the western parts of the city (Figs. 3 & 4) overlapped exactly with the areas in the map of income distribution and education in Mashhad [39]. The LL cluster overlapped with the high-income and high-education regions in the western part of the city, while the HH cluster matched the low-income and low-education areas in the East. These findings imply that socioeconomic status is related to PPRTIs, which is consistent with other studies [40–43]. In this connection, the effect of race, household income and living in poor and low-income areas examined on a block-by-block basis found that paediatric, pedestrian motor vehicle accidents occurred predominantly within low-income neighbourhoods, mostly African-American ones [44]. It should be noted that the relationship between socioeconomic status and pedestrian collision injuries is not limited to child pedestrians. A study conducted in San Francisco, USA to investigate the geographical correlation of pedestrian injury collisions with unemployment status of the regions discovered that there were higher pedestrian crashes for all age-groups in areas with higher unemployment rates [6]. Furthermore, the results of the study by Chakravarthy et al. [41] revealed that pedestrian crashes were 4 times more frequent in poor neighbourhoods but it was concluded that it did not appear to be due to the individual and family factors, age of the population, education, fluency in English or the density of population living in such neighbourhoods. In addition, Cubbin et al. [45] conducted a study on a sample of 472,364 persons in USA to determine individual and neighbourhood effects of external causes of injury (e.g., homicide, suicide, motor vehicle deaths, etc.). They showed that living in a neighbourhood characterized by low socioeconomic status increased the risk of external injury two folds, even after adjusting for individual demographic and socioeconomic characteristics [45]. Finally, it has been pointed out that the risk of PPRTIs is increased in poor areas, which is attributable to the physical environmental factors of poor neighbourhoods, such as traffic volume, urban and roads design, something which Soubhi calls “neighbourhood disadvantages” [46]. Figure 4 shows that the Abobargh neighbourhood, a crowded and poor neighbourhood with non-standard alleyways and streets in Mashhad [47, 48], is an HL outlier in terms of PPRTI distribution. Buildings and roads in this neighbourhood are also far from the principles of licensed urbanization [39] even if other neighbourhoods around this area are built in accordance with the latest urban standards.

Figure 5 shows that older children are more likely to experience accidents in western Mashhad, but younger children are more likely to be involved in accidents in the north-eastern part of the city. The different demographic composition of these two regions may be the cause. Since the type of accidents may be different for younger children than those involving older children, it seems that at least two different strategies for reducing the accidents should be planned for these two regions.

Our results show that 43% of all PPRTIs occurred on the street or along their sides, predominantly in neighbourhoods with low socioeconomic status in the south-eastern areas of the city. In this regard, our findings are consistent with the findings of the study by Lightstone et al. [24]. They showed that children were significantly more likely to experience accidents at local and collector streets of those census tracts with a larger number of families per census tract, i.e. a measure of household crowding and density [24].

### Limitations

In this study only the emergency calls related to PPRTIs were considered. Neither victims who left the accident scene without calling the EMS nor those transferred to hospitals by private vehicles were available. This limitation underestimates our results to some extent. However, this effect must be similar for the whole area, so it should not affect the identification of high-risk and low-risk areas. For future studies, we recommend researchers to link the hospitals and EMS data for obtaining the cases transferred to hospitals in other ways than by ambulance.

## Conclusions

Spatial analysis of PPRTIs in an urban area at different geographical scales provides supporting, reliable documentation that are useful for implementing and prioritize preventive strategies such as improvement of neighbourhoods and high-risk streets to lower the number of PPRTIs. At the macro level, it is recommended to redesign the texture of poor neighbourhoods leading to more standard roads with enough width proportional to the volume of traffic, especially high-risk roads. At the micro level, establishing safe passageways for pedestrians (such as bridges or underpasses) would allow people to completely avoid crossing streets at the same level as motorized vehicles and bicycles. Since about half of PPRTIs occur on the streets or at their sides, street crossings should be made safer, in particular with regard to the ways children move from one pavement to another, e.g., by erecting traffic sign posts at lower levels aimed at children. We also recommend examining the relationship between the number of safe passageways for pedestrians and PPRTIs in future studies. Effective educational methods for road safety measures, such as safe passages for children, need to be developed to educate them. The spatial age distribution of PPRTI victims is an important area to investigate to prioritize public health interventions.

## Data Availability

The datasets used and/or analysed during the current study are available from the corresponding author (B.K) on reasonable request.

## Abbreviations

PPRTIs: Paediatric, pedestrian road traffic injuries
HH: High-high
LL: Low-low
HL: High-low
LH: Low-high
WHO: World health organization
RTIs: Road traffic injuries
DALYs: Disability-adjusted life years
GIS: Geographical information systems
EMS: Emergency medical services
KML: Keyhole markup language
WGS: World Geodetic System
UTM: Universal Transverse Mercator
CL: Confidence Level
